# Differentiating non-lactating mastitis and malignant breast tumors by deep-learning based AI automatic classification system: A preliminary study

**DOI:** 10.3389/fonc.2022.997306

**Published:** 2022-09-15

**Authors:** Ying Zhou, Bo-Jian Feng, Wen-Wen Yue, Yuan Liu, Zhi-Feng Xu, Wei Xing, Zhao Xu, Jin-Cao Yao, Shu-Rong Wang, Dong Xu

**Affiliations:** ^1^ Department of Surgery, Hebei Provincial Hospital of Traditional Chinese Medicine, Shijiazhuang, China; ^2^ Department of Diagnostic Ultrasound Imaging and Interventional Therapy, The Cancer Hospital of the University of Chinese Academy of Sciences (Zhejiang Cancer Hospital), Hangzhou, China; ^3^ Department of Medical Ultrasound, Shanghai Tenth People’s Hospital, Ultrasound Research and Education Institute, Shanghai, China; ^4^ Department of Ultrasound Medicine, Yantai Affiliated Hospital of Binzhou Medical College, Yantai, China; ^5^ Institute of Basic Medicine and Cancer (IBMC), Chinese Academy of Sciences, Hangzhou, China

**Keywords:** deep-learning based AI automatic classification system, malignant breast tumors, nonlactating mastitis, plasma cell mastitis, granulomatous mastitis

## Abstract

**Objective:**

To explore the application values of deep-learning based artificial intelligence (AI) automatic classification system, on the differential diagnosis of non-lactating mastitis (NLM) and malignant breast tumors, *via* its comparation with traditional ultrasound interpretations and the following interpretation conclusions made by the sonographers with various seniorities.

**Methods:**

A total of 707 patients suffering from breast lesions (475 malignant breast tumors and 232 NLM), were selected from the following three medical centers, including Zhejiang Cancer Hospital, Hebei Province Hospital of Traditional Chinese Medicine, and Yantai Affiliated Hospital of Binzhou Medical University, and the time period was set from April 2020 to September 2021. All selected cases firstly accepted the routine breast ultrasound diagnosis, followed by the interpretations from a senior sonographer with more than 15 years of work experience, and an intermediate-aged sonographer with more than 5 years of work experience, independently. Meanwhile, a third physician also interpreted the same ultrasound images by deep learning–based AI automatic classification system, independent of the interpretation results from the previous two physicians. The kappa test was performed to evaluate the consistency between the conventional ultrasound interpretation results and pathological results interpreted from physicians with different working experiences.

**Results:**

In total, 475 cases of malignant breast tumors (512 nodules) and 232 cases of NLM (255 nodules) were pathologically diagnosed. The accuracy, sensitivity, and specificity of conventional ultrasound interpretations vary from different sonographers with different working experiences. The accuracy, sensitivity, and specificity for intermediate-aged sonographers and senior sonographers were 76.92% (590/767), 84.71% (216/255), and 73.95% (374/512) and 87.35% (670/767), 86.27% (220/255), and 87.89% (450/512), respectively (P<0.001). In contrast, if the threshold was set as 0.5, the accuracy, sensitivity, and specificity from deep learning–based AI automatic classification system were 83.00%, 87.20%, and 85.33%, separately, and the area under the curve was 92.6. The results of the kappa consistency test indicated that the diagnosis results from the image interpretations by senior physicians and deep-learning based AI automatic classification system showed high consistency with postoperative pathological diagnosis results, and the kappa values are 0.72 and 0.71, respectively, with the P-value of less than 0.001. In contrast, the consistency between the image interpretation results from intermediate-aged physicians with less working experience, and postoperative pathological diagnosis results, seemed to be relatively lower, with a kappa value of only 0.53 and P-value of less than 0.001.

**Conclusions:**

The deep learning–based AI automatic classification system is expected to become a reliable auxiliary way to distinguish NLM and malignant breast tumors due to its high sensitivity, accuracy, and specificity.

## Introduction

Generally, non-lactating mastitis (NLM) mainly includes plasma cell mastitis (PCM) (1) and granulomatous mastitis (GM) (2). It is a kind of inflammatory breast disease that occurs among the non-lactating women with the ages ranging between 30 and 40 years old (3, 4). It is relatively rare in clinical practice, and its rare incidence accounts for 1.41%–5.36% of the breast diseases in the same period, showing an increasing trend in recent years (5).

NLM belongs to a kind of rare, benign, and non-specific inflammatory breast disease and is usually misregarded as malignant breast tumors both clinically and radiologically (6, 7). Malignant breast tumors are a kind of common malignant tumors among women and show serious effects on both the physical and mental health of patients. The previous studies suggested that the 5-year survival for malignant breast tumors can be improved by more than 80% through early screening and diagnosis (8, 9). The breast imaging reporting and date system formulated by the American College of Radiology provided classification criteria for the ultrasound diagnosis of breast diseases (10). There are many clinical diagnostic methods, including nuclear magnetic resonance, tomography, three-dimensional reconstruction technology, and ultrasound examination (11–15). In some cases, due to the limited availability of medical imaging equipment, as well as the less working experience of physicians, misdiagnosis still occurs sometimes, leading to a patient’s failure to be diagnosed correctly (16). Conventional magnetic-resonance-imaging (MRI) scans have good soft tissue resolution, can image in multiple directions, and have no radiation. The diagnostic sensitivity is as high as 90%, but the specificity is only 50%–70% (17), and the cost is relatively high and the examination time is long, so it cannot be widely used in clinical work. Digital mammography has good spatial resolution, which is conducive to the observation of the overall shape of the lesion, and is the most specific for the detection of calcification, but the sensitivity of breast diagnosis decreases with the increase of gland density (18). Diagnosis is difficult due to atypical imaging manifestations, and the diagnosis of early small malignant breast tumors is also difficult (19). Ultrasound examination is real time, non-invasive, and sensitive to the breast examination of dense glands. It has now become an important means of routine examination of female breasts in China. However, considering some overlapped features between NLM and malignant breast tumors, and the fact that the interpretations of image features are also susceptible to the subjective experience of physicians, it is difficult to distinguish (20). In recent years, the emergence of many new technologies has made up for the shortcomings of conventional ultrasound diagnosis (21). An automated breast volume scanner (ABVS) is a fully automatic three-dimensional imaging scanner that can clearly display the information of the coronal plane of the lesion, but ABVS also has limitations: 1) it is not suitable for patients with large breasts, ulceration on the surface, partial depression, or an obvious protrusion of the tumor on the skin surface, and 2) it is impossible to superimpose technologies such as color Doppler and elastography like conventional ultrasound, and the diagnostic information is relatively simple (22). Elastography techniques mainly include strain elastography (SE) and shear wave elastography (SWE), which can qualitatively and quantitatively reflect the degree of softness and hardness of lesions in real time, but their diagnostic results are also affected by the operator’s experience, technology, and the depth of the lesion and size, as well as the influence of factors such as the region of interest of the selected lesion (23). Contrast-enhanced ultrasound can sensitively capture low-velocity blood flow signals and improve the detection rate of early malignant breast tumors, but its shortcomings are: 1) the results of contrast-enhanced imaging are affected by the injection method, instrument adjustment, contrast artifact, and lesion location; 2) benign and malignant breasts. The microcirculation state of the lesions overlaps, and angiography may not be able to distinguish it; and 3) the qualitative or quantitative criteria for evaluating the enhancement pattern of benign and malignant breast lesions are not unified (24). AI is a new technical science based on mathematics, computer science, etc., which researches and develops theories, methods, and application systems for simulating the extension and expansion of human intelligence (25). The earliest development of breast medical imaging AI technology is the computer-aided design (CAD) system. Traditional CAD is affected by artificial delineation and feature extraction (26), and its accuracy is not high. Deep learning can autonomously extract the fine features of massive images to achieve end-to-end learning. The convolutional neural network (CNN) is the most representative model of deep learning, which has excellent performance in the detection and classification of breast ultrasound images (27). AI performs advanced learning based on large data sets and has the advantages of fast calculation speed and strong repeatability. It is expected to become the right-hand assistant of sonographers in the future.

## Materials and methods

### Materials

A total of 707 patients diagnosed as breast nodules (475 malignant breast tumor, 232 NLM) were selected from three tertiary centers (Zhejiang Cancer Hospital, Hebei Province Hospital of Traditional Chinese Medicine, and Yantai Affiliated Hospital of Binzhou Medical University) with the period from April 2020 to September 2021. The gold standards were set up based on puncture or surgical pathological diagnosis, and we tried to compare the differences between the deep-learning AI automatic classification system and routine examinations from sonographers with different working experiences, in the diagnosis of NLM and malignant breast tumors, followed by the discussion of identification values for the deep-learning AI automatic classification system in distinguishing the above two diseases. All the enrolled patients were proven by histopathological results after biopsy, surgery, or both. The inclusion criteria were: (1) nodules were confirmed by puncture or surgical pathology as NLM or malignant breast tumor; (2) solid breast lesions or predominant solid lesions (cystic part <25%); (3) have not received treatments such as incision and drainage, intervention, radiotherapy, and chemotherapy; (4) breast lesions detected by conventional United States (US) and had complete transverse and longitudinal standard cross-sectional views and image data. The exclusion criteria were: (1) ultrasonic examination showed unclear horizontal and vertical standard sections; (2) unclear pathological diagnosis or benign breast nodules; and (3) patients suffering from breastfeeding mastitis. This study was approved by the hospital ethics committee, and the subjects signed an informed consent form before all examinations.

### Equipment and methods

#### Conventional ultrasound image interpretations

**Figure 1:**
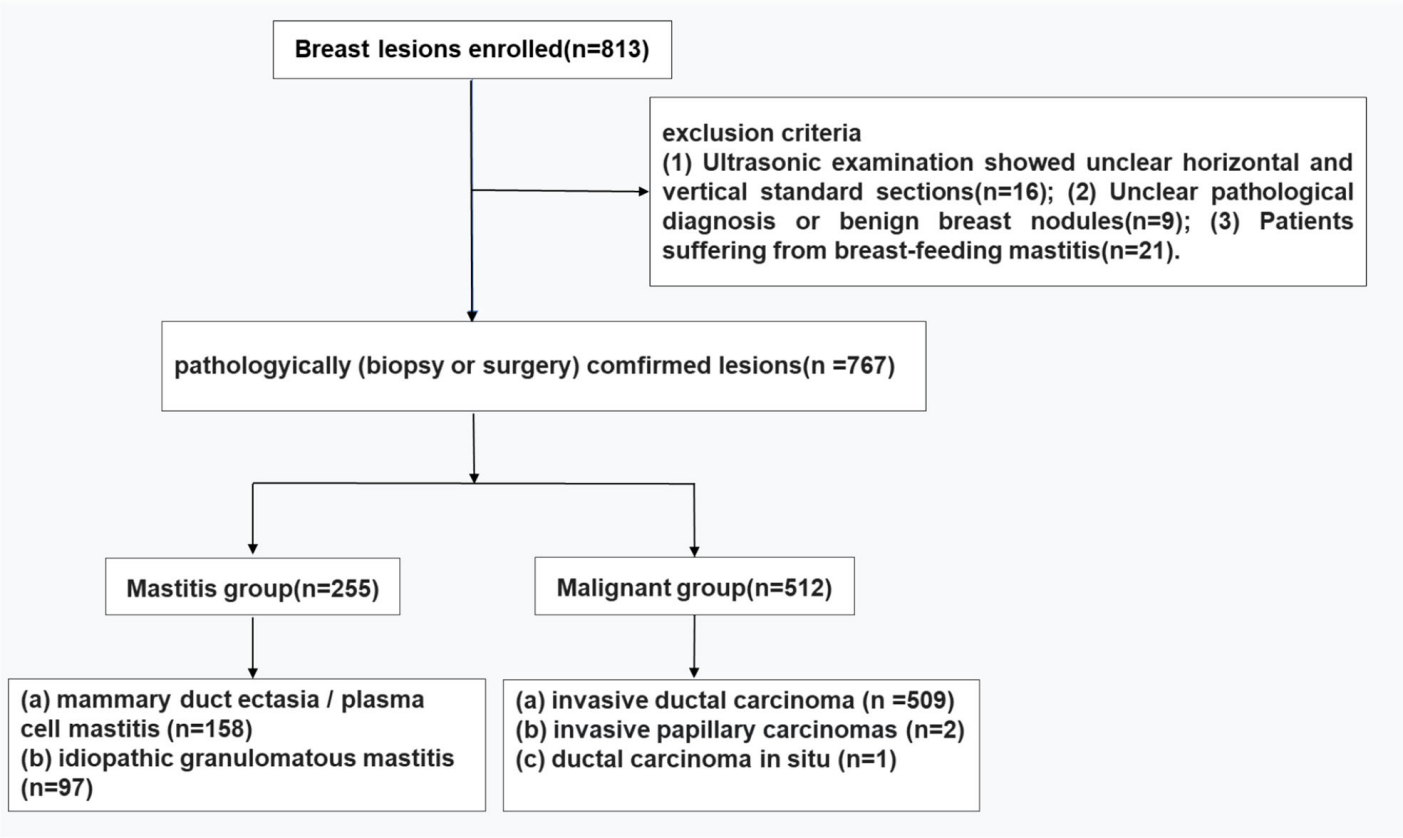
Flowchart for lesion selection.

The above three medical centers used different types of ultrasound diagnostic equipment (LOGIQ E9 for Hebei Provincial Hospital of Traditional Chinese Medicine and Zhejiang Cancer Hospital, and Toshiba Aplio500 for Yantai Affiliated Hospital of Binzhou Medical University) to perform the routine breast ultrasound examinations, with the probe model of L11 and frequency of 5~13 MHz. The resulting ultrasound images were interpreted by two sonographers who have been engaged in breast ultrasound diagnosis for many years (one senior physician with more than 15 years of work experience, and another with more than 5 years of work experience), separately. This study was investigated according to the breast imaging reporting and data system developed by the American College of Radiology, with the precondition of not knowing a patient’s personal clinical information and histopathological results (**Figure 1**).

#### Intelligence ultrasound image interpretations

The obtained ultrasound images were interpreted by a deep-learning based AI automatic classification system, which contains two parts including hardware and software. In terms of the software part, all methods were implemented in Python. CNNs have currently been applied widely in various fields (28–30), and the network in this study was based on Keras 2.1.5 and Tensorflow 1.6.0. The system runs under Ubuntu 16.04. For the hardware part, the system runs on Intel^®^ Xeon(R) CPU E5-2678 v3 @ 2.5 GHz×48 and NVIDIA TITAN V 12 GB Graphic Processing Unit (GPU).

To verify whether it works for all proposed methods above, we used the B-mode ultrasound database of breast nodules originated from multicenters, which contains the 1808 B-mode ultrasound pictures of breast nodules (767 nodules in total). After obtaining the original database, we firstly constructed a rectangular frame in the nodule area based on the segmentation results to obtain the images of the nodule, followed by the diagnosis as malignant breast tumor or NLM (marked as 0: malignant breast tumor, 1: NLM) by a physician with working experience for more than 5 years. A total of 1,250 pictures (512 nodules) for malignant breast tumors were obtained, of which 558 were NLM pictures (255 nodules). Considering the imbalances in the data amount among different categories, the method of data amplification was applied to increase the pictures of both NLM and malignant breast tumors, up to 30,000, respectively, meanwhile keeping the ratio as 1:1, adjusting all the picture sizes as 224 × 224. We chose to use the pretrained InceptionV3 deep CNN from the ImageNet data set for transfer learning, and, meanwhile, the replacement of the fully connected layer, SoftMax layer, and classification output layer of InceptionV3. Of all the collected samples, 70% were selected as the training data set and 20% as the verification data set, and the remaining 10% were used as the test data set (**Table 1**).

**Table 1:** The baseline of the patients included in the data set.

	Intermediate-aged physician (n = 767)	Senior physician (n = 767)	AI (n = 767, 1,250 pictures for malignant breast tumors, 558 were NLM pictures)		
**Pathologic stage**			Training data set	Verification data set	Test data set
IDC	509	509	357 (899 pictures)	101 (216 pictures)	51 (125 pictures)
IPC	2	2	1 (4 pictures)	1 (4 pictures)	0
IC	1	1	1 (2 pictures)	0	0
PCM	158	158	110 (246 pictures)	32 (77 pictures)	16 (62 pictures)
GM	97	97	68 (111 pictures)	19 (24 pictures)	10 (38 pictures)
**Age (y) (mean ± std)**	46.52 ± 10.29	46.52 ± 10.29	46.17 ± 10.30	47.04 ± 10.80	46.78 ± 10.56
**Nodule size**					
0–1.0 cm	272(35.46%)	272(35.48%)	190(35.38%)	55(35.95%)	27(35.06%)
1.0–2.0 cm	400(52.15%)	400(52.13%)	277(51.58%)	79(51.63%)	40(51.95%)
>2.0 cm	95(12.39%)	95(12.39%)	70(13.04%)	19(12.42%)	10(12.99%)
**Nodule location**					
RU	273(35.59%)	273(35.59%)	192(35.85%)	53(34.64%)	24(35.06%)
RM	54(7.04%)	54(7.04%)	38(7.08%)	9(5.88%)	5(6.49%)
RD	129(16.82%)	129(16.82%)	89(16.57%)	27(17.65%)	13(16.88%)
LU	192(25.03%)	192(25.03%)	132(24.67%)	38(24.83%)	19(24.69%)
LD	119(15.52%)	119(15.52%)	85(15.83%)	26(17.00%)	13(16.88%)

DC, invasive ductal carcinomas; IPC, invasive papillary carcinomas; IC, intraductal carcinoma; PCM, plasma cell mastitis; GM, granulomatous mastitis; RU, right-up lobe; RM, right-middle lobe; RD, right-down lobe; LU, left-up lobe; LD, left-down lobe.

**Figure 2:**
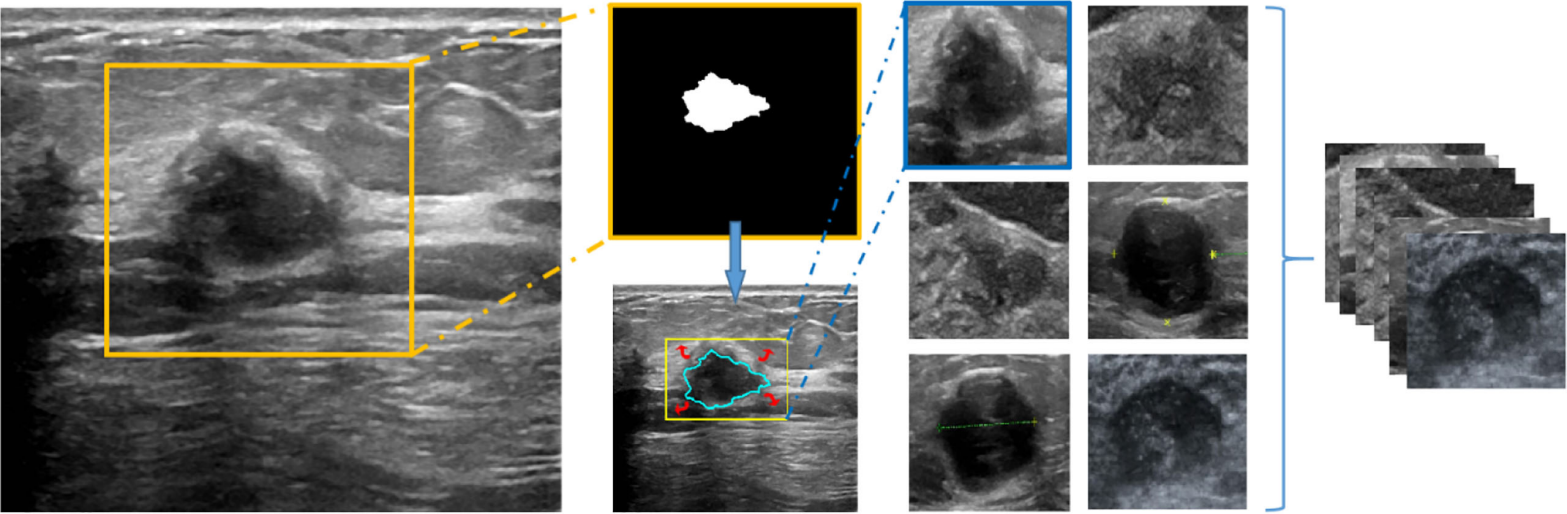
The working window of deep learning–based AI automatic classification system (the overall process of image preprocessing).

For each nodule, we extracted the outer box of the nodule area according to the doctor’s annotation of the nodule contour and then expanded it by 0.2 times in the up, down, left, and right directions to include the surrounding tissue and finally resized the cropped image to 224 × 224 and input it into the network. The entire procedure is shown in **Figure 2**.

**Figure 3:**
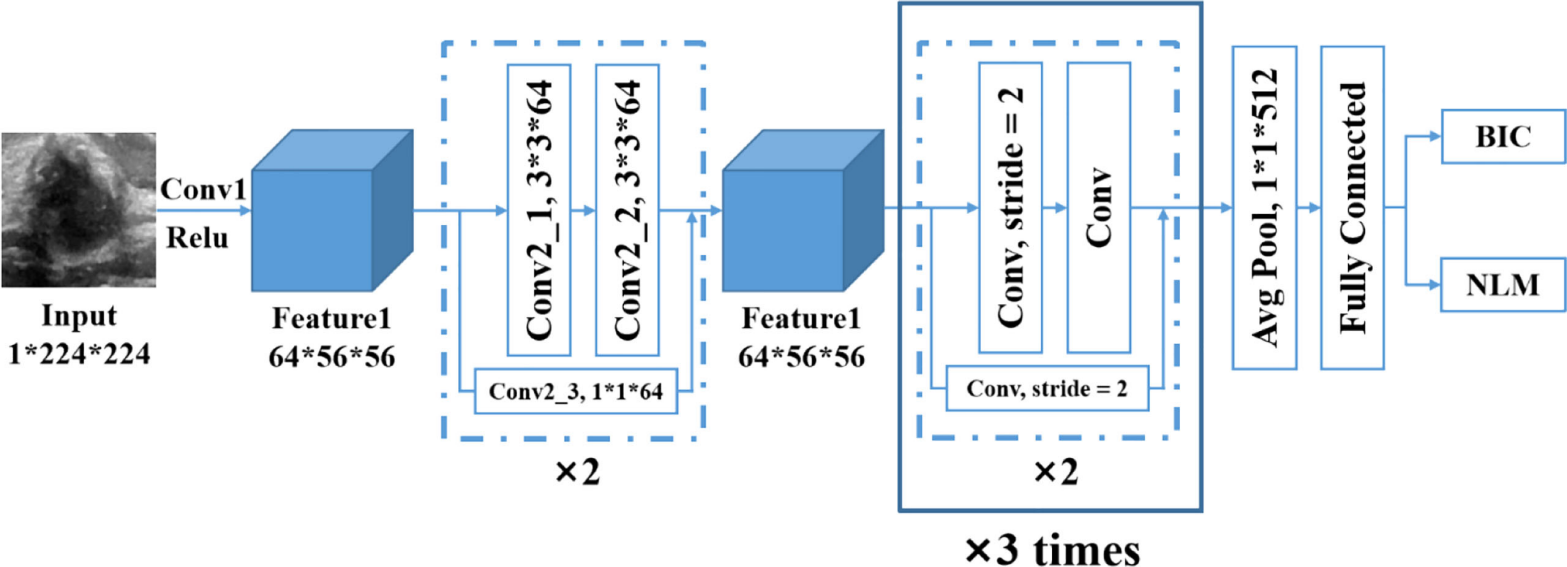
The network structure of ResNet-18.

ResNet is able to solve the problem of gradient disappearance in deep neural networks through identity mapping and accelerate the training process. The entire structure is shown in **Figure 3**.

### Statistical analysis

Statistical Product Service Solutions (SPSS) 26.0 was used for statistical analysis. Data were indicated as the form of x ± s and shown as the number of cases and percentages. The pathology results were set as the gold standard, and the consistency of pathological and interpretation results, from conventional ultrasound interpretation by two physicians with different working experiences, and deep learning–based AI automatic classification system were evaluated using the consistency test based on the kappa coefficient (31), followed by the calculations of the kappa coefficient, accuracy, sensitivity and specificity. The larger the kappa coefficient, the higher the consistency. The kappa value with more than 0.70 was interpreted as good consistency and the kappa value of 0.40~<0.70 as general and the kappa value with less than 0.40 as poor consistency. The inspection level (α) was 0.05.

## Results

### Pathological results

A total of 707 patients (767 nodules) were collected in this study, and the malignant breast tumors diagnosed by puncture or surgical pathology were 475 cases (512 nodules), of which invasive ductal carcinomas account for 509 cases, invasive papillary carcinomas for 2 cases, and intraductal carcinoma for 1 case. The remaining 232 cases were diagnosed as NLM (255 nodules), including 158 cases of PCM and 97 cases of granulomatous mastitis.

### Differential diagnosis of non-lactating mastitis and malignant breast tumors by sonographers with different working experiences and deep-learning based artificial intelligence automatic classification system

**Figure 4:**
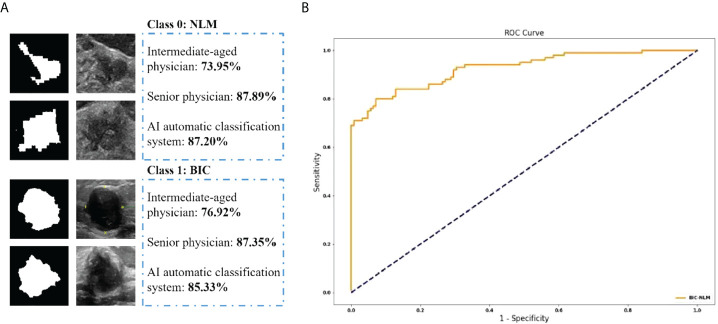
**(A)** The classification accuracy of physicians and AI automatic classification system in NLM and BIC. **(B)** The ROC curve of the proposed method.

Of the total 767 nodules, NLM diagnosed by pathological analysis account for 255 cases, and the remaining 512 cases were diagnosed as malignant tumors. The intermediate-aged sonographer diagnosed 354 cases as NLM and 413 cases as malignant tumors. Furthermore, NLM and malignant tumors, diagnosed by the senior sonographer, were 214 cases and 553 cases, respectively. In contrast, if the threshold value was set as 0.5, the model accuracy, sensitivity, and specificity of the deep-learning based AI automatic classification system were 85.33%, 83.00%, and 87.20%, respectively, similar to the diagnosis results from the senior sonographers [accuracy 87.35% (670/767), sensitivity 86.27% (220/255), and specificity 87.89% (450/512)] and higher than that of sonographers with intermediate-aged working experience [respectively 76.92% (590/767), 84.71% (216/255), and 73.95% (374/512)]. Furthermore, the area under the curve (AUC) was 0.926 (as shown in **Figure 4**).

Here, if we defined “mastitis” as positive cases, and “malignant breast tumor” as negative cases, then, the positive predicted values and negative predicted values of all NLM or malignant breast tumors, diagnosed by intermediate-aged/senior experienced physicians and the deep learning–based AI automatic classification system, were 61.02%, 78.01%, and 83.84% and 90.56%, 92.78%, and 86.51%, respectively. It suggested that the interpretations by senior physicians, or the deep learning–based AI automatic classification system, showed higher consistency with the postoperative pathological diagnosis results, with the kappa value of 0.72 and 0.71 and P-value of less than 0.001. In contrast, the results of image interpretations by physicians with intermediate-aged experience showed relatively lower consistency (its kappa value was 0.53 with the P-value of less than 0.001). For details, refer to **Tables 2**, **3**.

**Table 2:** The interpretation results by intermediate-aged/senior physicians based on working experience.

The interpretation ways		Pathological examination results	
		NLM (nodules)	Malignant breast tumors (nodules)
Intermediate-aged physician	NLM	216	138
	Malignant breast tumors	39	374
Senior physician	NLM	220	62
	Malignant breast tumors	35	450
AI	NLM	83	17
	Malignant breast tumors	16	109

NLM, non-lactating mastitis.

**Table 3:** The differential diagnosis of non-lactating mastitis and malignant breast tumors by both the deep learning–based AI automatic classification system and intermediate-aged/senior physicians.

Interpretation ways	Accuracy(%)	Sensitivity(%)	Specificity(%)	Positive predicted value (%)	Negative predicted value (%)	Kappa value	P-value
Intermediate-aged physician	76.92	84.71	73.95	61.02	90.56	0.53	<0.001
Senior physician	87.35	86.27	87.89	78.01	92.78	0.72	<0.001
AI automatic classification system	85.33	83.00	87.20	83.84	86.51	0.71	<0.001

## Discussions

NLM is a kind of inflammatory disease in the breast tissues of non-breastfeeding women. It generally belongs to benign lesions and is mainly characterized with duct dilatation and massive inflammatory cell infiltration and followed by the infiltrating hyperplasia of ducts and adjacent tissues in the late stage (32, 33). Its main clinical manifestations (34) include breast swelling and pain accompanied with festering, long-lasting unhealed and repeated attacks. Generally, it belongs to intractable diseases among benign breast diseases. Therefore, NLM is also clinically called “undead cancers”. The differentiation of this disease from malignant breast tumors can also lead to misdiagnosis easily. Malignant tumors in the breasts (35) are mostly invasive duct carcinoma, with the proliferation of fibrous tissues in the stroma. Thus, clinical manifestations are always presented as hard mass, an unclear boundary with the adjacent tissues, and poor mobility, along with some pains (36).

The ultrasound images of the above two diseases can show as hypoechoic or mixed-echo masses, obscure boundaries, irregular shapes, and heterogeneous internal echoes, along with or without strong echoes, and the partial attenuation of posterior echoes and CDFI show blood flow signals. The lesions of NLM become small along with the inhomogeneous internal echo; meanwhile, there are larger lesions existing in malignant breast tumors, along with necrosis liquefaction inside; the images of conventional ultrasound showed to be very similar, thus, to some degree, resulting in difficulties in differential diagnosis.

In recent years, with continuous update and improvement, AI-associated diagnosis technologies have been applied in the automatic segments and quick analysis of abnormal areas in tissues, as well as the quantification of lesions (37). Furthermore, AI can also be used for the accurate evaluations of detectable areas to reduce the medical mistakes caused by manual operations (38). AI-driven ultrasound is also becoming more and more mature, and its generated diagnosis results are also getting closer to the results of pathological diagnosis; the emergency of diagnosis by AI-driven ultrasound especially provides the beneficial supplements for early screening and benign/malignant assessments for high-risk diseases such as breast nodules and thyroid nodules (39).

In this study, the application of the deep learning–based AI automatic classification system reduced the probability of misreadings or misinterpretations, through the continuous input of cases, and followed with feature learning, lesion segmentation, and the extraction of features with multiple levels. However, considering the imbalances in the data amount among different categories, the method of data amplification was applied to increase the pictures of both NLM and malignant breast tumors, up to 30,000, respectively, meanwhile, keeping the ratio as 1:1 and adjusting all the picture sizes as 224 × 224.

We chose to use the pretrained InceptionV3 deep CNN from the ImageNet data set for the transfer learning and, meanwhile, the replacement of the fully connected layer, the SoftMax layer and classification output layer of InceptionV3. Then, the classification output layer was set up into two classes to generate the novel network model. The optimized algorithm uses the stochastic gradient descent method, and the hyperparameters of the model were set as follows: the initial learning rate was 0.001, the batch size was 128, the maximum epoch was 100, and the dropout probability was 0.5.

Furthermore, in this study, as for the AI analysis methods, the target areas were also preprocessed such as noise reduction, and enhancement/refinement for images, which improved the stability of AI interpretations, as well as the improvements in sensitivity, specificity, and accuracy. This research suggested that the differential diagnosis of NLM and malignant breast tumors by both the deep learning–based AI automatic classification system and the senior physicians with rich working experience showed high consistency with postoperative pathological diagnosis results. In contrast, the interpretation results from physicians with intermediate-aged working experience showed relatively lower consistency. When the threshold value was set as 0.5, the accuracy, sensitivity, and specificity of the model diagnosed by the deep learning–based AI automatic classification system were 85.33%, 83.00%, and 87.20%, respectively, and the AUC was 92.6. Both were close to the senior physicians [accuracy 87.35%, sensitivity 86.27%, and specificity 87.89%] and higher than those of middle-aged physicians [accuracy 76.92%, sensitivity 84.71%, and specificity 73.95%]. Since all the diagnostic results were compared with the pathological gold-standard diagnostic results, the consistency of kappa values between the middle-aged physicians/senior physicians/AI diagnosis and the pathological results was 0.53/0.72/0.71. The diagnostic efficiency of senior physicians/AI was significantly higher than that of the middle-aged physicians’ results. For junior physicians, the introduction of AI-assisted diagnostic reading function in the future will help improve the accuracy of diagnosis, and AI-assisted diagnosis prompts provide feasibility for the rapid ability improvement of junior physicians in the future. If the clinical application of the deep learning AI automatic classification system technology for joint diagnosis will significantly improve the diagnostic efficiency of middle-aged sonographers, it is suitable for the training of ultrasound residents and shortens the training period; it is suitable for the primary screening of nodules in physical examinations. It avoids missed diagnosis and unnecessary needle biopsy and reduces the risk of overdiagnosis; it can also greatly reduce the workload of clinicians, make hospitals of different levels achieve homogeneity, and improve the differential diagnosis rate of non-lactation mastitis and malignant breast tumors.

## Limitations

This study has several limitations: the amount of data in this study is small, the AI ​​automatic classification system does not have a large amount of data for in-depth research, and the results may have certain bias and error, which requires multicenter and large-scale research verification. Currently, the latest version only processes and analyzes static gray-scale ultrasound images and cannot perform an intelligent diagnosis of breast nodule elastography, color Doppler flow imaging, and other multimodalities; the real-time dynamic comprehensive scanning of ultrasound helps. However, the AI ​​automatic classification system technology can only analyze static ultrasound images, and the ultrasound characteristics of different sections of the same lesion are not completely consistent, which will affect the diagnostic results. Future research trends will focus on actively building open databases, optimizing the features of small data sets (fine annotation) on the basis of big data, and developing multimodal ultrasound AI that can effectively analyze dynamic videos, color Doppler images, and elastic images. It is a diagnostic tool that uses digital image processing technology to mark the ultrasound images accordingly and utilizes mammography, MRI, and pathological multimodal joint diagnosis to further improve the diagnostic performance, help clinicians deal with clinical problems more fully and freely, and provide breast imaging. The development of diagnostic disciplines provides new impetus and also shows the broad prospects of intelligent medical imaging in the future.

## Conclusions

The deep-learning based AI automatic classification system is expected to become a reliable auxiliary way to distinguish NLM and malignant breast tumors due to its high sensitivity, accuracy, and specificity.

## Data availability statement

The original contributions presented in the study are included in the article/supplementary material. Further inquiries can be directed to the corresponding authors.

## Ethics statement

The studies involving human participants were reviewed and approved by Hebei Medical Ethics Committee. The patients/participants provided their written informed consent to participate in this study.

## Author contributions

YZ and DX had full access to all of the data in the study and take responsibility for the integrity of the data and the accuracy of the data analysis. Concept and design: All authors. Acquisition, analysis, or interpretation of data: All authors. Drafting of the manuscript: YZ. Critical revision of the manuscript for important intellectual content: All authors. Statistical analysis: YZ, B-JF, and W-WY. Obtained funding: DX. Administrative, technical, or material support: DX and S-RW. Supervision: DX. All authors contributed to the article and approved the submitted version.

## Funding

This study is funded by the National Natural Science Foundation of China (No. 82071946) and Zhejiang Provincial Natural Science Foundation of China (No. LSD19H180001).

## Conflict of interest

The authors declare that the research was conducted in the absence of any commercial or financial relationships that could be construed as a potential conflict of interest.

## Publisher’s note

All claims expressed in this article are solely those of the authors and do not necessarily represent those of their affiliated organizations, or those of the publisher, the editors and the reviewers. Any product that may be evaluated in this article, or claim that may be made by its manufacturer, is not guaranteed or endorsed by the publisher.
